# Increased Visceral Adipose Tissue and Hyperinsulinemia Raise the Risk for Recurrence of Non-B Non-C Hepatocellular Carcinoma after Curative Treatment

**DOI:** 10.3390/cancers13071542

**Published:** 2021-03-26

**Authors:** Kenji Imai, Koji Takai, Takao Miwa, Toshihide Maeda, Tatsunori Hanai, Makoto Shiraki, Atsushi Suetsugu, Masahito Shimizu

**Affiliations:** Department of Gastroenterology/Internal Medicine, Gifu University Graduate School of Medicine, 1-1 Yanagido, Gifu 501-1194, Japan; koz@gifu-u.ac.jp (K.T.); takao.miwa0505@gmail.com (T.M.); toshi_z218@yahoo.co.jp (T.M.); hanai0606@yahoo.co.jp (T.H.); mshiraki-gif@umin.ac.jp (M.S.); asue@gifu-u.ac.jp (A.S.); shimim@gifu-u.ac.jp (M.S.)

**Keywords:** fasting immunoreactive insulin, non-viral hepatocellular carcinoma, recurrence risk factor, visceral adipose tissue

## Abstract

**Simple Summary:**

With the increasing prevalence of obesity and diabetes in most countries, the increase in hepatocellular carcinoma (HCC) associated with these factors has recently become a serious healthcare problem. HCC can often emerge in the non-cirrhotic liver among obese patients, and it might suggest that the conventional surveillance strategies for HCC, which is mainly targeted at cirrhotic patients with hepatitis B or C virus, might be insufficient in the overnutrition era. We tried to extract factors that affect recurrence-free survival in patients with non-viral HCC, among obesity and diabetes factors, together with the established recurrence risk factors, using a decision-tree analysis.

**Abstract:**

We investigated the factors affecting recurrence-free survival in patients with non-B non-C hepatocellular carcinoma (HCC) who received curative treatment. Decision-tree analysis was performed in 72 curative cases of non-B non-C HCC to extract the risk factors for recurrence. The reliability of the extracted risk factors was evaluated using the Kaplan–Meier method and the Cox proportional hazards model. The decision-tree analysis extracted three factors—visceral adipose tissue (VAT) index (VATI; <71 and ≥71 cm^2^/m^2^), which was the cross-sectional areas of VAT on the computed tomographic image at the umbilical level, normalized by the square of the height, fasting immunoreactive insulin (FIRI; <5.5 and ≥5.5 µU/mL), and alpha-fetoprotein (AFP; <11 and ≥11 ng/mL). The Cox proportional hazards model showed that VATI (hazard ratio (HR): 2.556, 95% confidence interval (CI): 1.191–5.486, *p* = 0.016), FIRI (HR: 3.149, 95% CI: 1.156–8.575, *p* = 0.025), and AFP (HR: 3.362, 95% CI: 1.550–7.288, *p* = 0.002) were all independent risk factors for HCC recurrence. Non-B non-C HCC patients with a higher VATI (≥71 cm^2^/m^2^) or higher FIRI (≥5.5 µU/mL) and AFP (≥11 ng/mL) if VATI was <71 cm^2^/m^2^ are prone to recurrence after curative treatment.

## 1. Introduction

Hepatocellular carcinoma (HCC) is one of the most common global malignancies [1]. HCC generally develops in patients with chronic liver damage due to various causative agents, such as persistent infection of hepatitis B virus (HBV) and hepatitis C virus (HCV), alcohol consumption, and obesity and diabetes-related metabolic disorders [2]. Among them, HBV and HCV are the most common etiological factors worldwide for developing HCC. However, the cases caused by hepatitis virus infection are declining due to advances in anti-viral therapy [3,4,5].

On the other hand, with the increasing prevalence of obesity and diabetes in most countries, the increase in HCC associated with these factors recently has become a serious healthcare problem [3,6]. Approximately 20% of nonalcoholic fatty liver disease (NAFLD), hepatic manifestations of obesity, and metabolic syndrome, present as nonalcoholic steatohepatitis (NASH), with a risk of progression to cirrhosis and HCC [7]. HCC mortality is increasing in the United States partly because the number of patients with obesity and diabetes-related metabolic disorders is also increasing [3]. In Japan, non-B non-C HCC, which is diagnosed as both HBs antigen- and HCV antibody-negative, is also rapidly increasing, whereas HCV-related HCC is decreasing [6].

Cirrhosis usually precedes the diagnosis of HCC in most patients; however, it is not always a prerequisite for HCC development, and this might apply particularly to non-B non-C HCC [7]. One of the mechanisms through which non-cirrhotic HCC develops in obese and diabetic patients might be the involvement of obesity-related oncogenic drivers, such as adipose-derived inflammation, lipotoxicity, and insulin resistance [7]. In the overnutrition era, detection of non-cirrhotic HCC at an early stage, among immense numbers of obese patients who have neither HBV nor HCV is a major challenge. However, useful clinical risk factors affecting the development of non-B non-C HCC are still not fully understood.

Recurrent HCC is more likely to occur than initial HCC [8]. Therefore, it is useful to analyze the recurrence risk factors of HCC after curative treatment to identify the high-risk groups for this malignancy. Several factors, including male sex, presence of cirrhosis, high alpha-fetoprotein (AFP) levels, large tumor foci, multiplicity of tumors, and pathologically high-grade atypia of tumor cells, were reported to increase the recurrence risk for HCC after curative treatment [9,10,11,12]. In addition to these established recurrence risk factors, several obesity-related factors for pathogenesis, including a higher level of homeostasis model assessment-insulin resistance (HOMA-IR), increases in the serum levels of leptin and oxidative stress, and excess accumulation of visceral adipose tissue (VAT), were also reportedly involved in the early recurrence of HCC after curative treatment [13,14,15,16].

In this study, we investigated the factors that would affect recurrence-free survival in patients with non-B non-C HCC who have received curative treatment. To prevent oversight in any recurrence risk factors, we comprehensively selected possible risk factors associated with liver functional reserve, the progression of primary HCC, and obesity and diabetes, and performed a decision-tree analysis, which is a useful method for identifying the risk classification of HCC recurrence [17,18].

## 2. Materials and Methods

### 2.1. Patients, Treatment, and Determination of Recurrence

HCC diagnosis was based on typical findings obtained by imaging modalities, including enhanced ultrasonography, dynamic computed tomography (CT), and dynamic magnetic resonance imaging (MRI). The selection criteria for the initial treatments were determined according to the guidelines for HCC by the Liver Cancer Study Group of Japan [19].

A total of 134 non-B non-C HCC patients were treated in our hospital between May 2006 and December 2019. Inclusion criteria were as follows—surgical resection or radiofrequency ablation (RFA) done as treatment for initial HCC and treatment effect diagnosed as curative by means of dynamic CT, MRI, or enhanced ultrasound examination. We excluded patients who could not follow-up at our hospital or suffered from other advanced cancers besides HCC. Finally, 72 patients were enrolled in this study. Patients were followed-up on an outpatient basis to assess imaging modalities, such as dynamic CT and MRI, which were performed every 3 months. Local recurrence was excluded in the present study, and only distant lesions were defined as recurrence. The recurrence-free survival time was defined as the interval from the date of the initial treatment to the date of recurrence, or until December 2019 for recurrence-free survivors. All study participants provided verbal informed consent, which was considered sufficient, as this study followed an observational research design that did not require new human biological specimens. The study design, including this consent procedure, was approved by the ethics committee of the Gifu University School of Medicine (ethical protocol code: 29–26).

### 2.2. Decision-Tree Analysis of Risk Factors Affecting the Recurrence of Non-B Non-C HCC

**Figure 1:**
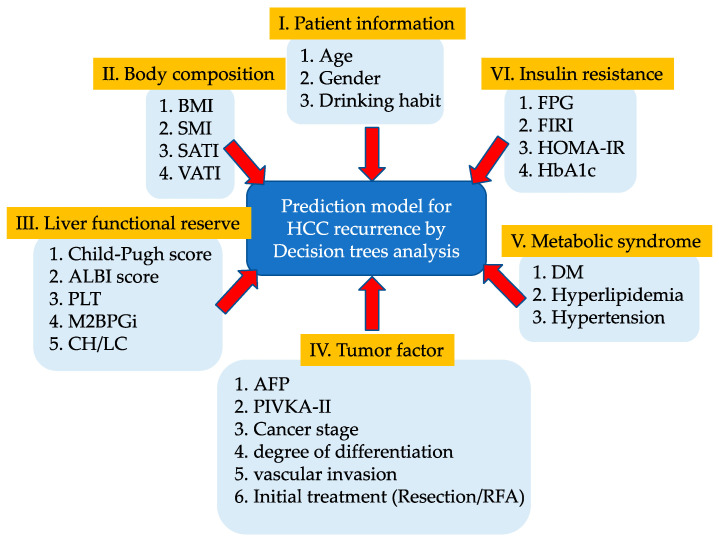
The outline of this study is based on the decision-tree analysis.

Decision-tree analysis [20] was performed to comprehensively measure as many possible risk factors for non-B non-C HCC recurrence as possible. We set the recurrence-free survival data, which consists of the observation time and the presence or absence of recurrence at the end of the observation, as an objective variable. On the other hand, the following factors were set as explanatory variables—(I) patients’ information (age, sex, and drinking habit (≥60 g/day for men and ≥40 g/day for women in ethanol amount)); (II) body composition—body mass index (BMI), skeletal muscle index (SMI), subcutaneous adipose tissue (SAT) index (SATI), and VAT index (VATI); (III) liver functional reserve—Child-Pugh score, albumin–bilirubin (ALBI) score, platelet count, Mac2 binding protein glucosylation isomer (M2BPGi), and the presence of cirrhosis; (IV) tumor factor—AFP, proteins induced by vitamin K absence or antagonist-II (PIVKA-II), clinical cancer stage, the degree of differentiation, the presence of vascular invasion, and initial treatment (resection or RFA); (V) metabolic syndrome—the presence of diabetes mellitus (DM), hyperlipidemia, and hypertension; and (VI) insulin resistance—fasting plasma glucose (FPG), fasting immunoreactive insulin (FIRI), HOMA-IR, and HbA1c. The outline of this study is shown in Figure 1.

The cross-sectional areas of the muscle (cm^2^) at the L3 level of the CT image were normalized by the square of the height (m^2^) to obtain SMI (cm^2^/m^2^). Similarly, the cross-sectional areas of SAT and VAT (cm^2^) at the umbilical point were normalized by the square of the height (m^2^) to obtain the SATI and VATI (cm^2^/m^2^), respectively [21]. We used the SYNAPSE VINCENT software (Fujifilm Medical, Tokyo, Japan) to measure the cross-sectional areas of these tissues.

### 2.3. Statistical Analyses

We used ‘rpart’ package (version 4.1–15) in R for conducting the decision-tree analysis. The settings for decision-tree analysis were as follows—method was ‘exp’; minimum number of observations was 25; complexity parameter was 0.02; other settings left the default value. Recurrence-free survival time was estimated using the Kaplan–Meier method. Differences between curves were evaluated using the log-rank test. The Cox proportional-hazards model was also used to confirm that the recurrence risk factors extracted using the decision-tree analysis would independently affect recurrence-free survival. The Tukey method was used as a post-hoc test to counteract the problem of multiple comparisons between the groups, divided by decision-tree analysis. Statistical significance was defined as *p* < 0.05. All statistical analyses were performed using R version 4.0.0 (R Foundation for Statistical Computing, Vienna, Austria; http://www.R-project.org/, accessed on 26 March 2021).

## 3. Results

### 3.1. Baseline Characteristics and Laboratory Data of Enrolled Patients

The baseline characteristics and laboratory data of the 72 patients (49 males and 23 females; average age, 72.6 years) prior to the curative treatment for initial non-B non-C HCC are shown in Table 1. The median BMI and VATI were 24.7 kg/m^2^ and 55.1 cm^2^/m^2^, respectively. Etiology was attributed to NASH (66.7%), alcohol (25.0%), and other factors (8.3%). Of the enrolled patients, 75% were diagnosed with cirrhosis and 61.1% had diabetes.

### 3.2. Possible Risk Factors Affecting Recurrence-Free Survival of Non-B Non-C HCC Patients

**Figure 2:**
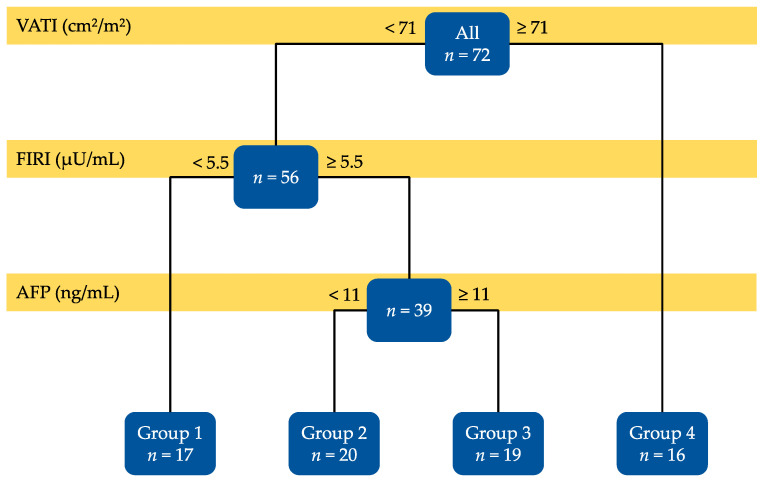
The result of the decision-tree analysis on factors predicting non-B non-C HCC recurrence.

The results of the decision-tree analysis are shown in Figure 2. Among the factors expected to be involved in the development of non-B non-C HCC (Figure 1), VATI (<71 and ≥71 cm^2^/m^2^), FIRI (<5.5 and ≥5.5 µU/mL), and AFP (<11 and ≥11 ng/mL) were identified as significant predictors for recurrence. The enrolled patients were also divided into four groups based on these three factors, which were associated with recurrence-free survival rates. The patients with VATI ≥71 cm^2^/m^2^ had the worst recurrence-free survival (Group 4, *n* = 16). Among the remaining 56 patients, patients with <5.5 µU/mL of FIRI had the best recurrence-free survival (Group 1, *n* = 17). Among the remaining 39 patients who showed lower VATI (<71 cm^2^/m^2^) but higher FIRI (≥5.5 µU/mL), patients with <11 ng/mL of AFP (Group 2, *n* = 20) had better recurrence-free survival than those with ≥11 ng/mL (Group 3, *n* = 19). The recurrence-free survival rates of the enrolled patients are described in the next section.

The Cox proportional hazards model also showed that the three factors extracted using the decision-tree analysis including VATI (≥71 vs. <71 cm^2^/m^2^; hazard ratio (HR): 2.556, 95% confidence interval (CI): 1.191–5.486, *p* = 0.016), FIRI (≥5.5 vs. <5.5 µU/mL; HR: 3.149, 95% CI: 1.156–8.575, *p* = 0.025), and AFP (≥11 vs. <11 ng/mL; HR: 3.362, 95% CI: 1.550–7.288, *p* = 0.002), were all independent risk factors for HCC recurrence (Table 2). We also evaluated the same analyses stratified by gender. For males, the decision-tree analysis demonstrated that VATI (<66 and ≥66 cm^2^/m^2^) and FIRI (<5.5 and ≥5.5 µU/mL) were identified as significant predictors for recurrence (Supplemental Figure S1). The Cox proportional hazards model showed that VATI and AFP were independent risk factors (Supplemental Table S1). For females, neither the decision-tree analysis nor the Cox proportional hazards model extracted any significant predictor for HCC recurrence as the sample size was too small (Supplementary Table S2).

### 3.3. Recurrence-Free Survival Rates of the Enrolled Patients

**Figure 3:**
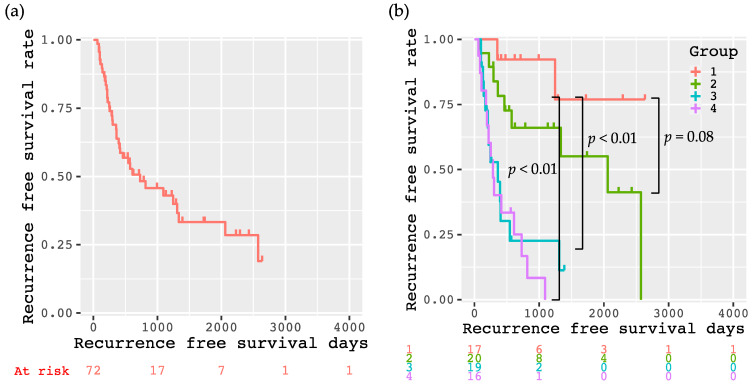
Kaplan–Meier curves for recurrence-free survival after curative treatment in all participants (**a**), and divided into four groups according to the decision-tree analysis (**b**). Group 1 meets the following conditions: VATI < 71 cm^2^/m^2^ and FIRI < 5.5 µU/mL; Group 2: VATI < 71 cm^2^/m^2^, FIRI ≥ 5.5 µU/mL, and AFP < 11 ng/mL; Group 3: VATI < 71 cm^2^/m^2^, FIRI ≥ 5.5 µU/mL, and AFP ≥ 11 ng/mL; and Group 4: VATI ≥ 71 cm^2^/m^2^.

The Kaplan–Meier method showed that the 1-, 3-, and 5-year recurrence-free survival rates of all enrolled patients were 63.8%, 43.0%, and 33.3%, respectively (Figure 3a). These rates for each respective group divided according to the decision-tree analysis were 92.3%, 92.3%, and 76.9% (Group 1); 78.3%, 66.1%, and 55.1% (Group 2); 45.3%, 22.7%, and 11.3% (Group 3); and 40.2%, 0%, and 0% (Group 4), respectively. Patients in Group 1 had significantly better recurrence-free survival than those in Group 3 (*p* < 0.01) and Group 4 (*p* < 0.01), as per the log-rank test results. The recurrence-free survival of Group 1 also tended to be better than that of Group 2 (*p* = 0.08, Figure 3b). When comparing the differences of baseline demographic and clinical characteristics among the four groups, there were significant differences in BMI, SMI, and VATI (Table 3); however, no significant differences were found in the other factors including AFP and FIRI. The level of AFP (<11 and ≥11 ng/mL) was not involved in the recurrence free survival of the patients in Group 1 (Supplemental Figure S2a). Additionally, the levels of AFP (<11 and ≥11 ng/mL) and FIRI (<5.5 and ≥5.5 µU/mL) did not affect the recurrence free survival of the patients in Group 4 (Supplementary Figure S2b,c).

The 1-, 3-, and 5-year overall survival rates of all enrolled patients were 92.1%, 72.3%, and 47.0%, respectively (Supplementary Figure S3a). Although there were not significant differences in overall survival among the four groups (*p* = 0.087), patients in Group 1 and 2 tended to have longer survival than those in Group 3 and 4. (Supplementary Figure S3b).

## 4. Discussion

Obesity is a serious healthcare problem worldwide [22,23]. A large number of studies revealed a link between obesity and the development of HCC [22,24]. The number of patients with NAFLD/NASH are reportedly increasing, and HCC can often emerge in the non-cirrhotic liver among obese NAFLD/NASH patients [7,25]. In this study, none of the factors associated with liver functional reserve were risk factors for non-B non-C HCC recurrence. These include the Child-Pugh score, ALBI score, platelet count, M2BPGi, and the presence of cirrhosis, which significantly increase the risk of developing virus-associated HCC [10]. These facts suggest that the conventional surveillance strategies for HCC mainly targeted at cirrhotic patients with HBV or HCV might be insufficient in the overnutrition era. Therefore, finding suitable obesity-related HCC risk factors based on the consideration of the mechanism that links obesity to liver carcinogenesis is urgently needed for screening the development of virus-negative HCC.

The results of the decision-tree analysis conducted in this cohort clearly showed that the excess accumulation of VAT (≥71 cm^2^/m^2^) was the strongest risk factor for non-B non-C HCC recurrence. Accumulation of VAT, which involves the recruitment of macrophages, is considered to play a critical role in liver carcinogenesis [7,26]. VAT leads to an altered pattern of adipokine secretion, which is involved in systemic inflammation, tumor growth, and angiogenesis [7,27]. Insulin resistance or hyperinsulinemia, which are often seen in patients with a high volume of VAT, also promotes the development of HCC through the activation of tumor growth and angiogenesis [7]. Consistent with these mechanisms, we observed in the present study that the patients in Group 1 who have lower VAT (<71 cm^2^/m^2^) and FIRI (<5.5 µU/mL) exhibited the best prognosis without experiencing HCC recurrence.

A high BMI is reportedly associated with an increased risk of HCC development [28]. However, our findings on how the accumulation of VAT predicts the recurrence of non-B non-C HCC suggest that in addition to BMI, more attention should be paid to body fat distribution, in order to screen patients who have a high risk of developing HCC. The physiological functions of VAT and SAT are quite different. Fat cells in SAT act as a buffer or sink for circulating free fatty acids and triglycerides. SAT might also be used as an energy source when receiving chemotherapy for HCC, and its rapid depletion predicts the poor survival of these patients [21,29]. Therefore, a body fat distribution assessment might be useful in predicting high-risk and poor prognosis groups for HCC.

Hyperinsulinemia was the second strongest predictor of non-B non-C HCC recurrence, whereas the FPG, HOMA-IR, and HbA1c values were not. This suggests that insulin itself is more deeply involved in liver carcinogenesis than insulin resistance and serum glucose levels. Our findings were consistent with the results of previous reports showing that insulin and insulin-stimulating drugs, such as sulfonylurea, significantly increase the risk of HCC [30,31]. According to the meta-analysis, metformin use was associated with a 50% reduction in the risk of developing HCC, whereas sulfonylurea or insulin use was associated with a 62% and 161% increase in the risk of HCC, respectively [30]. Insulin can stimulate the growth of HCC cells and promote vascular invasion of the tumor [7]. Together with these previous studies, this study might imply that we should treat diabetic patients with potential risk of HCC development with anti-diabetic medications that do not lead to hyperinsulinemia. In the present study, higher levels of both FIRI and AFP were associated with an increased risk of non-B non-C HCC recurrence. as high AFP levels reportedly indicate more severe vascular invasion and the existence of minute lesions that cannot be detected by imaging modalities [10], microvascular invasion or the growth of microtumors might be accelerated by hyperinsulinemia, especially in these patients.

This study has several limitations. First, it was a retrospective, single-center study, and the sample size was small as compared to those of the previous studies, showing the usefulness of decision-tree analysis for detecting a high-risk group for HCC recurrence [17,18]. For instance, according to the documentation that estimates sample sizes for the comparison of survival curves by the log-rank statistic [32], we need a sample size of about 200 if we want to compare a patient group with two years median recurrence free survival to one with three years. Second, this study did not focus on initial HCC, but on recurrent HCC after curative treatment. Thus, it might include a certain number of intrahepatic metastasis cases, which can impair the reliability of assessing the risk of hepatocarcinogenesis itself. We should analyze cases of cancer-free liver diseases other than HBV or HCV-related hepatitis, in order to purely estimate the risk of non-viral hepatocarcinogenesis. However, at the moment, such analysis seems difficult because risk factors of non-viral HCC are not clearly defined and we cannot screen the immense number of patients in the high-risk group of obese who neither have HBV nor HCV. Third, VATI presented as the strongest risk factor in this study is not a simple and safe index because a CT must be performed to obtain VATI. It is not realistic to perform a CT for all obese people. To overcome these limitations, a prospective study involving a larger number of non-B non-C patients without HCC enrolled from several centers should be conducted in the future.

## 5. Conclusions

The decision-tree analysis showed that non-B non-C HCC patients with a higher VATI (≥71 cm^2^/m^2^), and higher serum levels of insulin (≥5.5 µU/mL) and AFP (≥11 ng/mL) are prone to recurrence after curative treatment. Since the number of obese people is currently increasing rapidly worldwide, obesity or diabetes-related risk factors (VATI and FIRI), together with the established risk factors (AFP) for HCC recurrence might be useful in screening obese patients with a higher risk of HCC development and recurrence. Future studies enrolling a larger number of patients are important to verify the effects of these factors on the development of non-B non-C HCC.

## Figures and Tables

**Table 1:** Baseline demographic and clinical characteristics of the enrolled patients on primary non-B non-C HCC.

Variables	(*n* = 72)
Sex (male/female)	49/23
Age (years)	72.6 ± 9.1
Etiology (NASH/alcohol/others)	48/18/6
BMI (kg/m^2^)	24.7 ± 3.5
SMI (cm^2^/m^2^)	45.5 ± 6.9
SATI (cm^2^/m^2^)	46.8 ± 28.0
VATI (cm^2^/m^2^)	55.1 ± 26.5
Drinking habit (yes/no)	18/54
DM (yes/no)	44/28
Hypertension (yes/no)	39/33
Hyperlipidemia (yes/no)	17/55
FPG	119.3 ± 36.4
FIRI	15.0 ± 25.0
HOMA-IR	5.1 ± 11.2
HbA1c (%)	6.4 ± 1.3
Child-Pugh score (5/6/7/8/9/10)	49/16/4/1/1/1
ALBI score	−2.57 ± 0.44
Underlying liver disease (CH/LC)	18/54
M2BPGi	1.9 ± 1.8
Stage (I/II/III/IV)	20/13/28/0
AFP (ng/mL)	878 ± 3175
PIVKA-II	18,269 ± 80,487
Degree of differentiation (well/moderate/poor/unknown)	6/33/7/26
Capsule formation (yes/no/unknown)	32/11/29
Vascular invasion (yes/no)	9/63
Initial treatment (resection/RFA)	47/25

Values are presented as mean ± standard deviation. NASH, nonalcoholic steatohepatitis; BMI, body mass index; SMI, skeletal muscle index; SATI, subcutaneous adipose tissue index; VATI, visceral adipose tissue index; DM, diabetes mellitus; FPG, fasting plasma glucose; FIRI, fasting immunoreactive insulin; HOMA-IR, homeostasis model assessment-insulin resistance; HbA1c, hemoglobin A1c; ALBI score, albumin and bilirubin score; CH, chronic hepatitis; LC, liver cirrhosis; M2BPGi, Mac2 binding protein glucosylation isomer; AFP, alpha-fetoprotein; PIVKA-II, proteins induced by vitamin K absence or antagonist-II; and RFA, radiofrequency ablation.

**Table 2:** Univariate and multivariate analyses of possible risk factors affecting recurrence-free survival of non-B non-C HCC by Cox proportional hazards model.

Variables	Univariate Analysis		Multivariate Analysis	
	HR (95%CI)	*p* Value	HR (95%CI)	*p* Value
Sex (male vs. female)	0.398 (0.699–2.795)	0.343		
Age (years)	0.999 (0.968–1.031)	0.940		
Drinking habit (yes vs. no)	1.440 (0.711–2.919)	0.311		
DM (yes vs. no)	1.923 (0.964–3.835)	0.063		
Hyperlipidemia (yes vs. no)	1.100 (0.503–2.407)	0.811		
Hypertension (yes vs. no)	1.248 (0.658–2.370)	0.498		
VATI (≥71 vs. <71 (cm^2^/m^2^))	2.718 (1.381–5.349)	0.004	2.556 (1.191–5.486)	0.016
Child-Pugh score	1.104 (0.787–1.548)	0.568		
PLT (× 10^4^/mL)	1.001 (0.961–1.043)	0.947		
FIRI (≥5.5 vs. <5.5 (µU/mL))	2.805 (1.065–7.388)	0.037	3.149 (1.156–8.575)	0.025
LC (yes vs. no)	1.422 (0.623–3.242)	0.403		
AFP (≥11 vs. <10 (ng/mL))	2.171 (1.131–4.166)	0.020	3.362 (1.550–7.288)	0.002
Degree of differentiation (moderate vs. well) (poor vs. well)	2.794 (0.646–12.08) 3.471 (0.626–19.26)	0.169 0.155		
Vascular invasion (yes vs. no)	2.237 (0.972–5.152)	0.058		
Initial treatment (RFA vs. Resection)	0.923 (0.467–1.824)	0.818		

HR, hazard ratio; DM, diabetes mellitus; VATI, visceral adipose tissue index; PLT, platelet count; FIRI, fasting immunoreactive insulin; LC, liver cirrhosis; AFP alpha-fetoprotein; and RFA, radiofrequency ablation.

**Table 3:** Baseline demographic and clinical characteristics of the enrolled patients with primary non-B non-C HCC divided into four groups according to the decision-tree analysis

Variables	Group 1 (*n* = 17)	Group 2 (*n* = 20)	Group 3 (*n* = 19)	Group 4 (*n* = 16)	*P* Value
Sex (male/female)	12/5	13/7	12/7	12/4	0.873
Age (years)	72.2±12.3	72.3 ± 10.5	71.4 ± 6.6	74.8 ± 5.7	0.729
Etiology (NASH/alcohol/others)	12/5/0	14/4/2	9/8/2	13/1/2	0.105
BMI (kg/m^2^)	21.4 ± 2.7	25.0 ± 2.1	25.6 ± 3.6	26.9 ± 3.3	<0.001
SMI (cm^2^/m^2^)	40.0 ± 3.6	44.8 ± 7.6	48.1 ± 6.6	48.9 ± 5.6	<0.001
SATI (cm^2^/m^2^)	34.5 ± 18.4	48.7 ± 21.4	50.5 ± 29.1	53.1 ± 38.6	0.209
VATI (cm^2^/m^2^)	34.2 ± 14.2	50.9 ± 14.0	48.2 ± 17.4	90.7 ± 23.8	<0.001
Drinking habit (yes/no)	5/12	4/16	8/11	1/15	0.093
DM (yes/no)	8/9	11/9	13/6	12/4	0.327
Hypertension (yes/no)	8/9	8/12	13/6	10/6	0.265
Hyperlipidemia (yes/no)	4/13	2/18	6/13	5/11	0.356
FPG	113.6 ± 43.8	117.1 ± 24.1	113.1 ± 28.6	135.3 ± 46.1	0.250
FIRI	3.9 ± 0.9	22.8 ± 33.0	19.8 ± 30.8	11.7 ± 13.0	0.124
HOMA-IR	1.1 ± 0.6	6.7 ± 10.3	7.3 ± 17.1	4.7 ± 8.5	0.389
HbA1c (%)	6.3 ± 1.5	6.4 ± 1.1	6.2 ± 1.0	7.0 ± 1.6	0.277
Child-Pugh score (5/6/7/8/9/10)	12/3/1/0/0/1	12/6/2/0/0/0	12/4/1/1/1/0	13/3/0/0/0/0	0.686
ALBI score	−2.52 ± 0.44	–2.56 ± 0.45	–2.58 ± 0.47	−2.64 ± 0.45	0.890
Underlying liver disease (CH/LC)	7/10	4/16	4/15	3/13	0.372
M2BPGi	1.4 ± 0.6	1.6 ± 0.9	2.7 ± 3.1	1.8 ± 1.3	0.408
Stage (I/II/III/IV)	2/10/3/2	8/9/3/0	4/6/8/1	3/6/7/0	0.141
AFP (ng/mL)	1686 ± 3782	5.6 ± 2.3	541 ± 997	1510 ± 5379	0.331
PIVKA-II	12375 ± 48613	3242 ± 8843	18528 ± 72059	40205 ± 140137	0.609
Degree of differentiation (well/moderate/poor/unknown)	2/7/2/6	3/6/1/10	1/10/1/7	0/10/3/3	0.419
Capsule formation (yes/no/unknown)	9/2/6	7/3/10	7/3/9	9/3/4	0.914
Vascular invasion (yes/no)	3/14	0/20	3/16	3/13	0.258
Initial treatment (resection/RFA)	11/6	11/9	13/6	12/4	0.662

Values are presented as mean ± standard deviation. NASH, nonalcoholic steatohepatitis; BMI, body mass index; SMI, skeletal muscle index; SATI, subcutaneous adipose tissue index; VATI, visceral adipose tissue index; DM, diabetes mellitus; FPG, fasting plasma glucose; FIRI, fasting immunoreactive insulin; HOMA-IR, homeostasis model assessment-insulin resistance; HbA1c, hemoglobin A1c; ALBI score, albumin and bilirubin score; CH, chronic hepatitis; LC, liver cirrhosis; M2BPGi, Mac2 binding protein glucosylation isomer; AFP, alpha-fetoprotein; PIVKA-II, proteins induced by vitamin K absence or antagonist-II; and RFA, radiofrequency ablation.

## Data Availability

The data presented in this study are available on request from the corresponding author.

## Supplementary Materials

The following are available online at https://www.mdpi.com/article/10.3390/cancers13071542/s1. Figure S1: The result of the decision-tree analysis on factors predicting non-B non-C HCC recurrence for men. Figure S2. Kaplan–Meier curves for recurrence-free survival after curative treatment in Group 1 divided into the cutoff value (11 ng/mL) of AFP (a), those in Group 4 divided into the cutoff value (11 mg/mL) of AFP (b), and those in Group 4 divided into the cutoff value (5.5 μU/mL) of FIRI (c); Figure S3. Kaplan–Meier curves for overall survival after curative treatment in all participants (a), and divided into four groups according to the decision-tree analysis (b). Group 1 meets the following conditions: VATI < 71 cm^2^/m^2^ and FIRI < 5.5 μU/mL; Group 2: VATI < 71 cm^2^/m^2^, FIRI ≥ 5.5 μU/mL, and AFP < 11 ng/mL; Group 3: VATI < 71 cm^2^/m^2^, FIRI ≥ 5.5 μU/mL, and AFP ≥ 11 ng/mL; Group 4: VATI ≥ 71 cm^2^/m^2^. Table S1: Univariate and multivariate analyses of possible risk factors for men affecting recurrence-free survival of non-B non-C HCC by Cox proportional hazards model. Table S2. Univariate and multivariate analyses of possible risk factors for women affecting recurrence-free survival of non-B non-C HCC by Cox proportional hazards model.
