# Supplementary material for: Increased Visceral Adipose Tissue and Hyperinsulinemia Raise the Risk for Recurrence of Non-B Non-C Hepatocellular Carcinoma after Curative Treatment
Source: Cancers (Basel). 2021 Mar 26;13(7):1542. doi: 10.3390/cancers13071542 (PMC8036481; doi:10.3390/cancers13071542)
Supplement: Supplementary file 1 [file cancers-13-01542-s001.pdf]

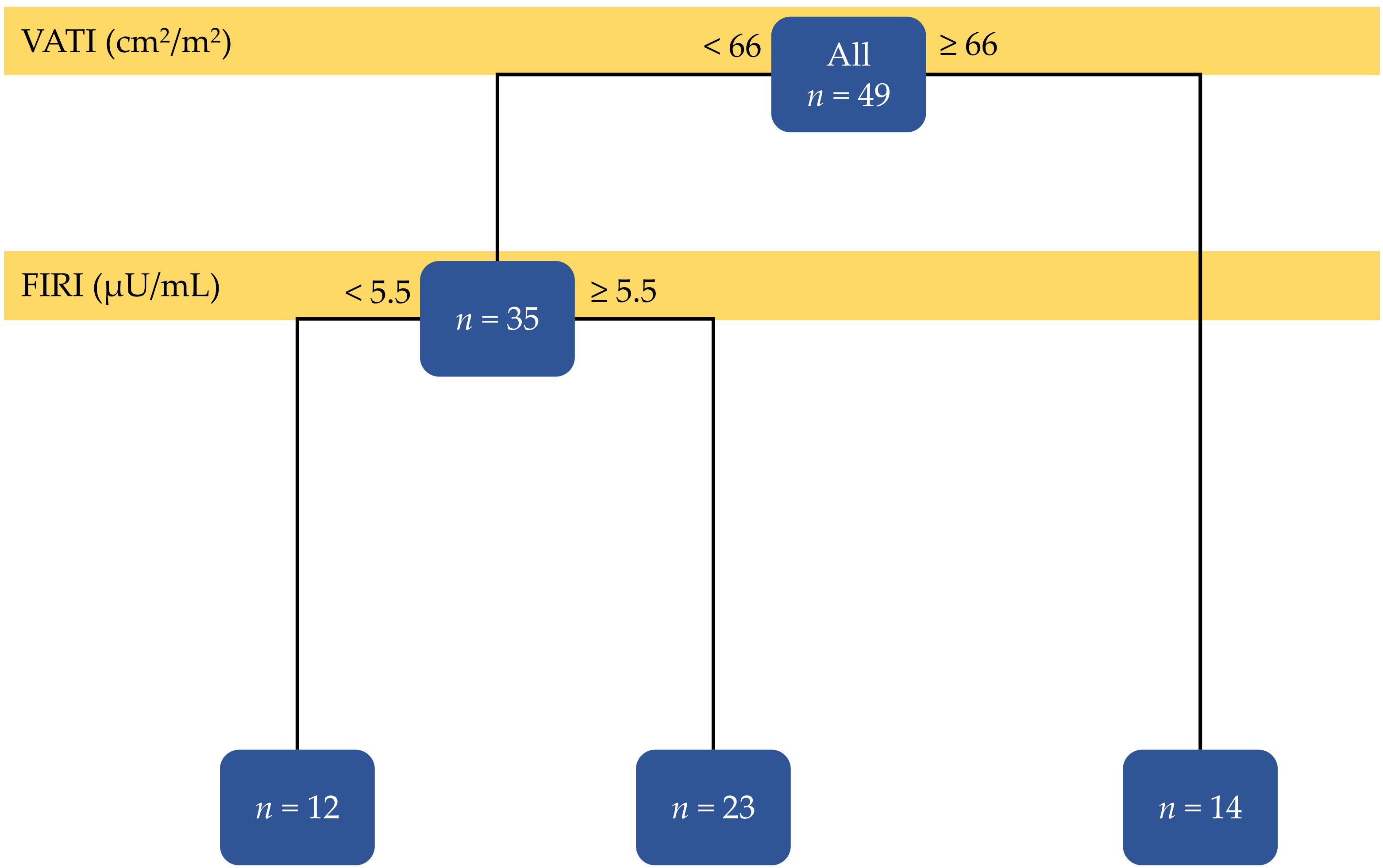

Figure S1. The result of the decision-tree analysis on factors predicting non-B non-C HCC recurrence for men.

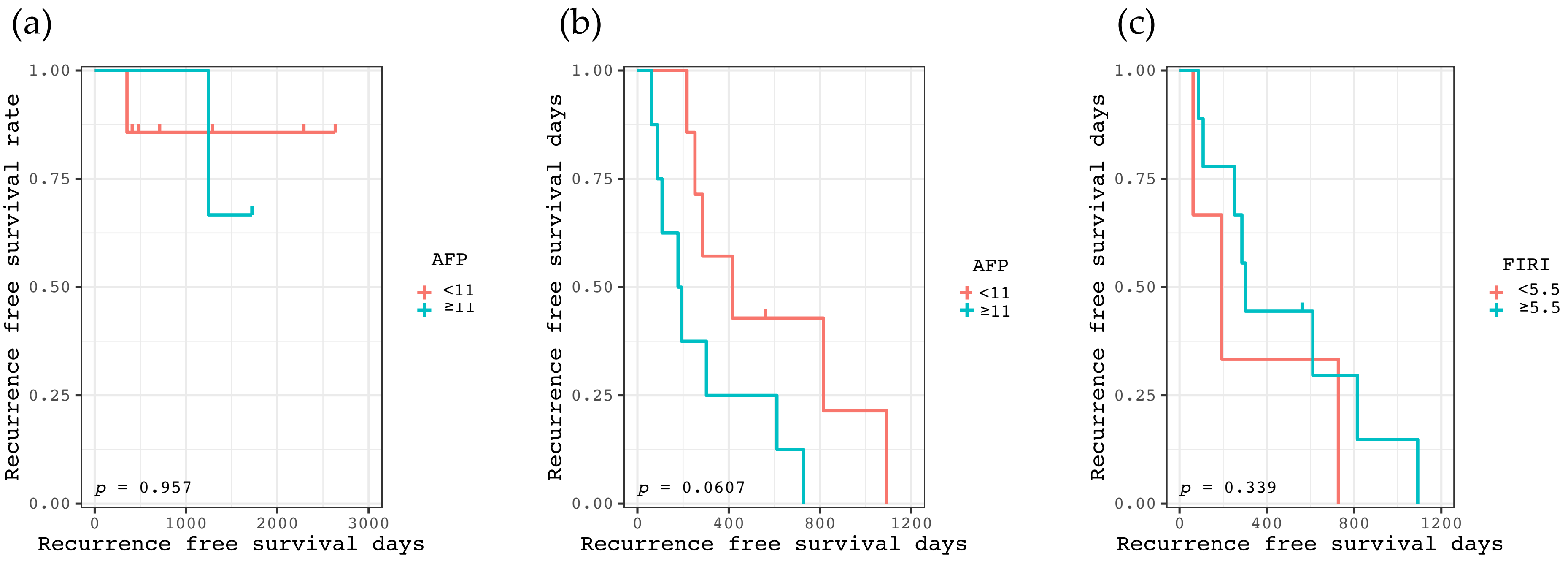

Supplemental Figure 2. Kaplan–Meier curves for recurrence-free survival after curative treatment in Group 1 divided into the cutoff value (11 ng/mL) of AFP (a), those in Group 4 divided into the cutoff value (11 mg/mL) of AFP (b), and those in Group 4 divided into the cutoff value (5.5 μU/mL) of FIRI (c).

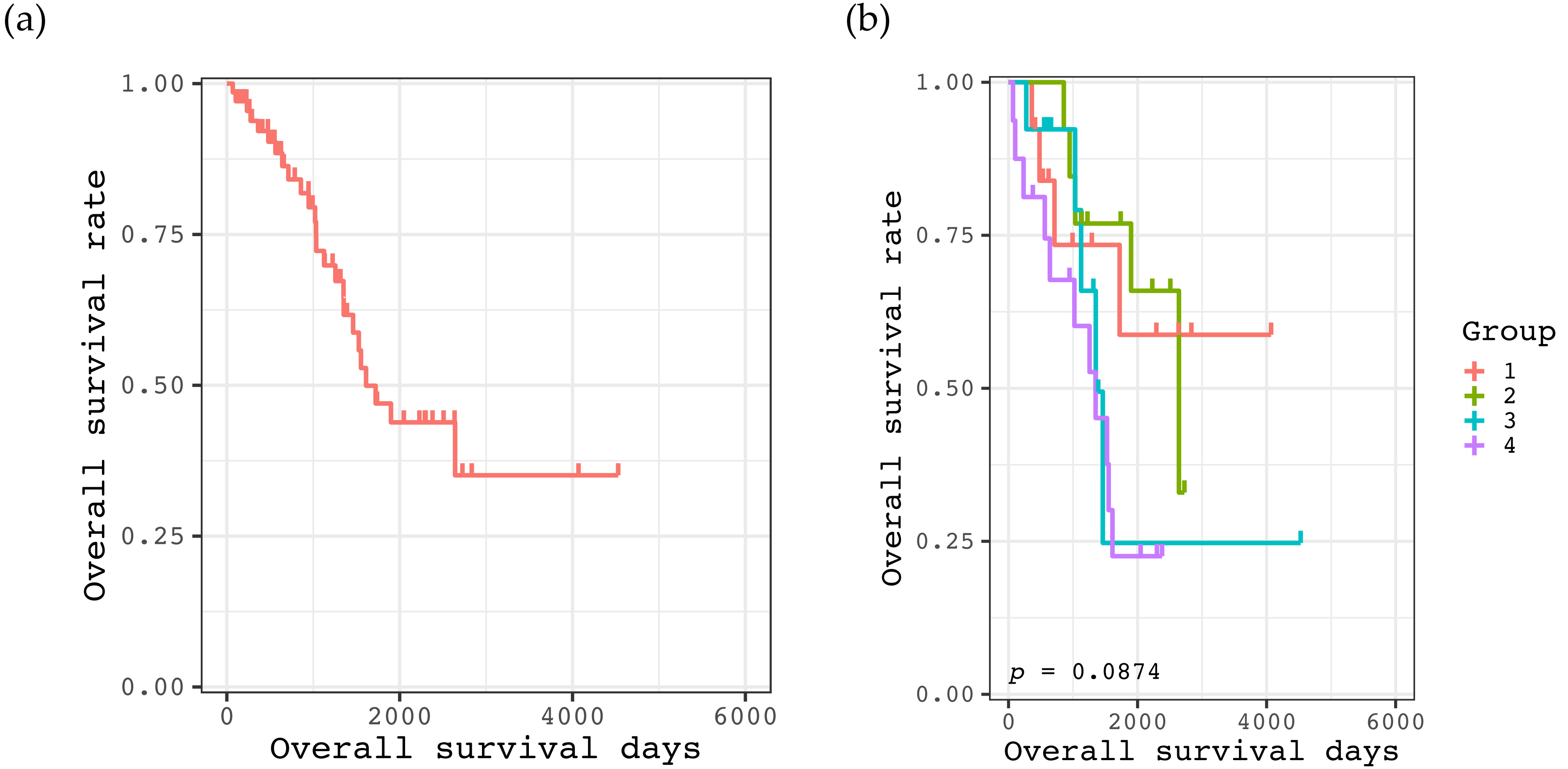

Supplemental Figure 3. Kaplan–Meier curves for overall survival after curative treatment in all participants (a), and divided into four groups according to the decision-tree analysis (b). Group 1 meets the following conditions: VATI <71 cm^2/m^2 and FIRI <5.5 μU/mL; Group 2: VATI <71 cm^2/m^2, FIRI ≥5.5 μU/mL, and AFP <11 ng/mL; Group 3: VATI <71 cm^2/m^2, FIRI ≥ 5.5 μU/mL, and AFP ≥11 ng/mL; Group 4: VATI ≥71 cm^2/m^2.

Table S1. Univariate and mutivariate analyses of possible risk factors for men affecting recurrence-free survival of non-B non-C HCC by Cox proportional hazards model

Variables	Univariate Analysis		Multivariate Analysis	
	**HR (95%CI)**	***p* value**	**HR (95%CI)**	***p* value**
Age (years)	0.997 (0.956–1.040)	0.905		
Drinking habit (yes vs. no)	1.185 (0.534–2.629)	0.676		
DM (yes vs. no)	1.791 (0.793–4.042)	0.161		
Hyperlipidemia (yes vs. no)	1.221 (0.510–2.921)	0.654		
Hypertention (yes vs. no)	0.849 (0.388–1.858)	0.682		
VATI (≥71 vs. <71 [cm^2/m^2])	3.350 (1.439–7.800)	0.005	3.027 (1.293–7.083)	0.010
Child-Pugh score	0.975 (0.562–1.690)	0.927		
PLT (× 10^4/mL)	1.012 (0.957–1.070)	0.672		
FIRI (≥5.5 vs. <5.5 [μU/mL])	3.106 (0.889–10.86)	0.076		
LC (yes vs. no)	0.936 (0.374–2.340)	0.887		
AFP (≥11 vs. <10 [ng/mL])	3.355 (1.524–7.387)	0.003	3.109 (1.407–6.871)	0.005
Degree of differentiation				
(moderate vs. well)	1.544 (0.347–6.860)	0.563		
(poor vs. well)	1.179 (0.158–8.784)	0.872		
Vascular invasion (yes vs. no)	1.694 (0.673–4.265)	0.263		
Initial treatment (RFA vs. Recection)	0.906 (0.353–2.328)	0.838		

HR, hazard ratio; DM, diabetes mellitus; VATI, visceral adipose tissue index; PLT, platelet count; FIRI, fasting immunoreactive insulin; LC, liver cirrhosis; AFP. alpha-fetoprotein; RFA, radiofrequency ablation

Table S2. Univariate and mutivariate analyses of possible risk factors for women affecting recurrence-free survival of non-B non-C HCC by Cox proportional hazards model

Variables	Univariate Analysis		Multivariate Analysis	
	HR (95%CI)	*p* value	HR (95%CI)	*p* value
Age (years)	1.004 (0.956–1.054)	0.877		
Drinking habit (yes vs. no)	3.759 (0.438–32.2)	0.227		
DM (yes vs. no)	2.200 (0.591–8.195)	0.240		
Hyperlipidemia (yes vs. no)	0.545 (0.070–4.244)	0.562		
Hypertention (yes vs. no)	3.427 (0.899–13.07)	0.071		
VATI (≥71 vs. <71 [cm^2/m^2])	1.855 (0.555–6.199)	0.315		
Child-Pugh score	1.293 (0.812–2.061)	0.279		
PLT (× 10^4/mL)	0.977 (0.902–1.058)	0.567		
FIRI (≥5.5 vs. <5.5 [μU/mL])	2.469 (0.535–11.39)	0.247		
LC (yes vs. no)	4.103 (0.525–32.06)	0.178		
AFP (≥11 vs. <10 [ng/mL])	1.088 (0.342–3.465)	0.886		
Degree of differentiation				
(moderate vs. well)	9703 (0–Inf)	0.999		
(poor vs. well)	2819 (0–Inf)	0.999		
Vascular invasion (yes vs. no)	10.5 (0.951–115.7)	0.056		
Initial treatment (RFA vs. Resection)	1.192 (0.376–3.778)	0.765		

HR, hazard ratio; DM, diabetes mellitus; VATI, visceral adipose tissue index; PLT, platelet count; FIRI, fasting immunoreactive insulin; LC, liver cirrhosis; AFP. alpha-fetoprotein; RFA, radiofrequency ablation
